# Cross shelf benthic biodiversity patterns in the Southern Red Sea

**DOI:** 10.1038/s41598-017-00507-y

**Published:** 2017-03-27

**Authors:** Joanne Ellis, Holger Anlauf, Saskia Kürten, Diego Lozano-Cortés, Zahra Alsaffar, Joao Cúrdia, Burton Jones, Susana Carvalho

**Affiliations:** 1 King Abdullah University of Science and Technology (KAUST), Red Sea Research Center, Division of Biological and Environmental Science and Engineering, Thuwal, 23955-6900 Saudi Arabia; 2 Environmental Protection Department, Saudi Aramco, Dhahran 31311 Saudi Arabia

## Abstract

The diversity of coral reef and soft sediment ecosystems in the Red Sea has to date received limited scientific attention. This study investigates changes in the community composition of both reef and macrobenthic communities along a cross shelf gradient. Coral reef assemblages differed significantly in species composition and structure with location and depth. Inner shelf reefs harbored less abundant and less diverse coral assemblages with higher percentage macroalgae cover. Nutrient availability and distance from the shoreline were significantly related to changes in coral composition and structure. This study also observed a clear inshore offshore pattern for soft sediment communities. In contrast to the coral reef patterns the highest diversity and abundance of soft sediment communities were recorded at the inshore sites, which were characterized by a higher number of opportunistic polychaete species and bivalves indicative of mild disturbance. Sediment grain size and nutrient enrichment were important variables explaining the variability. This study aims to contribute to our understanding of ecosystem processes and biodiversity in the Red Sea region in an area that also has the potential to provide insight into pressing topics, such as the capacity of reef systems and benthic macrofaunal organisms to adapt to global climate change.

## Introduction

The Red Sea is a confined water body by the Arabian Peninsula and African mainland, stretching from 30°N to 12°40′N over 1900 km and reaching a maximum width of 335 km^1^. Its latitude extension and arid continental setting make the Red Sea experience high rates of evaporation, a wide range of seasonal shallow water temperature regimes from 18 to 32 °C and above global ocean average saline conditions in the range of 37 to 42 psu^2, 3^. There is limited coastal runoff of freshwater into the Red Sea therefore the water loss of 2 m year^−1^ by evaporation is replenished almost entirely by oceanic waters from the Mediterranean via the Suez Canal and from the Indian Ocean by the narrow Bab-al-Mandab Strait^4, 5^. These combined conditions make the Red Sea one of the world’s warmest and most saline habitats in which extensive coral reefs occur, and therefore also a region of increasing interest for scientists working on climate change.

Much of the information on the Red Sea lies within technical reports, and there is relatively little information available to researchers using modern channels^6^. The Red Sea region is also relatively inaccessible due to permitting regulations and therefore remains a poorly studied system^6^. Further, the vast majority of published research for coral assemblages originates from an approximately 6 km stretch of the coastline in the far northern Red Sea near the Gulf of Aqaba while the literature on the soft sediment benthos of the Red Sea has been reported as very limited. Despite these research limitations, the Red Sea has long been recognized ecologically as one of the world’s biodiversity hotspots and an area of high endemism^7–9^. More than 364 scleractinian coral species (hard corals) have been recorded; of which 5.8% were suggested to be endemic^9^. Additionally, new reports of endemic scleractinian corals support that high levels of speciation exist in the Red Sea^10^. A few early studies have provided initial records on the abundance of soft sediment macrobenthos in the region and also provided support for high levels of soft sediment benthic species diversity^11, 12^. There is still a current lack of understanding of the general ecology of the Red Sea despite the region’s high biodiversity^6^. Increasingly anthropogenic impacts are affecting the ecology of the Red Sea region including rapid human population growth and urbanization, coastal construction, tourism, reef over-usage^13^ and destructive fisheries^14^. Thus, marine resource management priorities and the urgent need for monitoring programs have been identified as of high importance for the Red Sea region^15^.

As part of a recent initiative between the Red Sea Research Centre and Saudi Aramco, regional assessments have been undertaken to characterise the Red Sea environment for marine spatial planning efforts and to provide baseline information for future monitoring programs. In this study, we assessed regional biodiversity patterns from both coral reef and soft sediment data collected across a shelf gradient in the Farasan Islands. Spatial patterns in the distribution and abundance of species have been reported to change significantly over cross shelf gradients^16^, however the underlying drivers of species change across these gradients has not previously been fully assessed. There has been an increasing focus worldwide on regional assessments of biodiversity that can link the role of environmental variables in structuring community composition^17^. The understanding of regional biodiversity patterns and species distribution relative to their habitat is fundamental^18^ providing data that could inform science-based management approaches^6^. Most diversity studies across coastal shelves have, however, focused on single taxonomic groups, such as corals, with very few studies assessing multi-taxon diversity patterns in a marine context (exceptions include^19, 20^). Multi-taxon studies addressing the dynamic structuring of ecological communities have been beneficially utilized in testing and implementing high-quality conservation strategies^19^. Within this study, we present results of coral reef surveys following a longitudinal gradient at the Farasan Islands, ranging from inshore to offshore locations. We also present cross shelf biodiversity data collected from soft sediment macrobenthic assemblages investigating environmental variables that influence species distribution. The specific aims of this paper are to: (a) provide baseline information on both coral reef and soft sediment biodiversity patterns for the Red Sea region where there is limited information published; (b) to assess soft sediment and coral reef diversity patterns across a shelf gradient; and (c) to identify the role of environmental variables in structuring coral reef and soft sediment benthic communities. The present study aims to contribute to a better understanding of ecosystem processes and current biodiversity in the southern Red Sea region, an area that has the potential to provide insight into pressing topics such as the capacity of reef systems and benthic macrofaunal organisms to adapt to global climate change. This is, to our knowledge, the first synoptic study of benthic soft sediment faunal species richness and benthic coral reef diversity in the Red Sea that also relates biodiversity patterns to environmental and hydrographic variables.

## Material and Methods

### Study area

**Figure 1:**
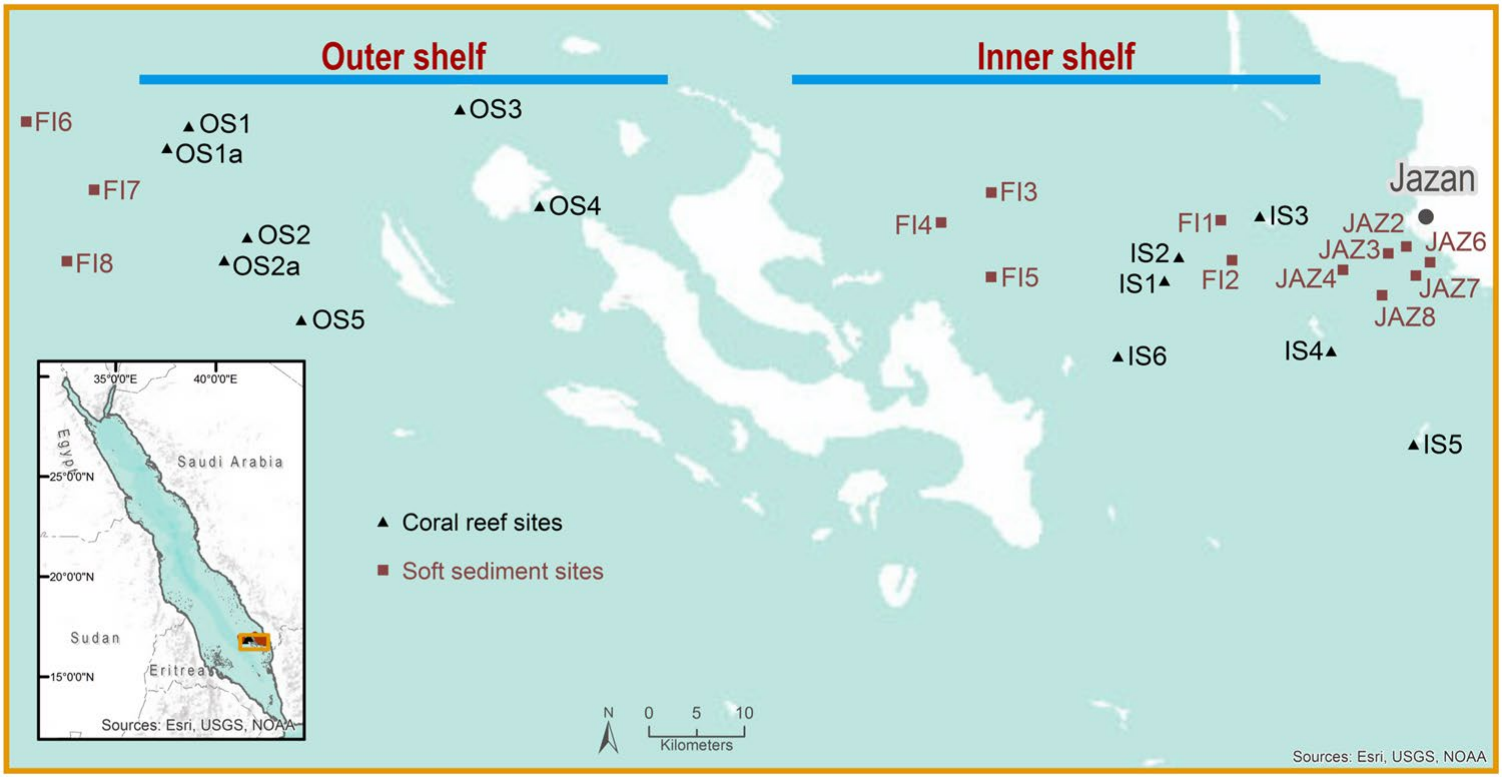
Map showing the location of the reef and soft sediment sites sampled across the Farasan shelf in the southern Red Sea. This figure was created using the ArcGIS software (ArcMap 10.3.1) by ESRI, Inc. (ESRI.com), GEBCO, DeLorme, US Geological Survey (USGS) and the National Oceanic and Atmospheric Administration (NOAA) photo library.

Soft sediment and reef surveys were conducted in the southern Red Sea to assess cross shelf gradient biodiversity patterns. The study area included sites around the Jazan City region and the Farasan Island Marine Sanctuary (Fig. 1). The wider Jazan province has a population of approximately 1.5 million and covers an area of 40,000 km^2^. Jazan City is an urbanized area with agricultural activities also present in the region. Three of the main islands at the Farasan Island Marine Sanctuary are permanently inhabited (Farasan, 369 km^2^; Sajid, 109 km^2^; Qummah, 14.3 km^2^) with a total population of approximately 4,500 inhabitants. Despite being considered a Marine Sanctuary, most of the inhabitants engage in fishing and agricultural activities. The Farasan Island plateau slopes gently over a distance of 120 km never reaching water depths greater than 200 m^21^.

### Reef survey

#### Quantitative surveys of benthic reef community

Coral reefs were assessed in February and September 2014. A total of 11 reef locations, six in the inner shelf (IS; IS1, IS2, IS3, IS4, IS5, IS6) and five in the outer shelf (OS; OS1, OS2, OS3, OS4, OS5), were surveyed using a 1 m wide photo quadrate belt-transect (Fig. 1). The transect line ideally followed the depth horizons at 3–5 m and 8–10 m. At some locations only the shallow transect was completed because a limestone reef foundation did not exist at deeper locations. Three replicate 20 m by 5 m transects comprised the surveyed 60 m^2^ along a horizontal stretch of slope at each reef site and depth. Along each transect, a photo (1 m^2^) was taken every two meters with a Canon G16 16 megapixel digital camera in Nauticam housing. The benthic community was assessed based on 90 benthic groups including scleractinian coral genera, abundant soft coral genera and other benthic community groups. Quantification and identification of benthic groups along each transect replicate utilized the software Coral Point Count with Excel extensions (CPCe^22^), with 40 randomly distributed points per 1 m^2^ frame. Coral identification was undertaken at family level for Fungidae or genus level for other Scleractinia following Veron^23^ despite the current revisions on coral’s taxonomy being undertaken worldwide. Counted points were averaged for each site and depth to calculate percentage cover of each group including corals (calcifying corals including Millipora), soft corals, sponges, coralline algae, turf algae, macroalgae and the abiotic components sand, rock and rubble. Forty points were randomly distributed on each substrate image and the features underlying the points user-identified. Overall, per photo-transect, benthic community composition was analyzed in terms of number of Operational Taxonomic Units (OTUs), cover of corals (both soft and scleractinian), sponges, hydroids and other invertebrates (e.g. bivalves, echinoderms), algae (subdivided by the following functional groups: macroalgae, turf, coralline algae) (see Table 1 for main benthic categories). Water samples for nutrients and Chlorophyll *a* were also collected from surface waters at each reef site (see water sampling methods below).

**Table 1:** Main categories used to define benthic community composition.

Benthic Component	Category
Abiotic	Rock
	Sand
Algae	Algal assemblage
	Coralline algae
	Halimeda
	Macroalgae
	Sargassum
	Turf algae
Coral	Soft coral
	Scleractinian
Dead coral	Dead coral
	Dead coral with algae
	Rubble
Invertebrates	Anemones
	Ascidians
	Bivalve
	Hydroids
	Sponge
	Zoanthids
	Other invertebrates
Seagrass	Seagrass

#### Size frequency distribution of corals

Negatively skewed size-frequency distributions in coral populations have been associated with unfavorable environmental conditions. Using the same photoquadrat data, we compared the size-frequency distributions between near shore and offshore reefs in the four most abundant genera (*Acropora*, *Echinopora*, *Porites* and *Galaxea*) to explore the occurrence of any differences in the skewness of the size distribution of the corals as a proxy for different environmental conditions. The colony diameter (maximum and minimum) was measured using the software ImageJ to estimate the colony area (projected surface area). Colony area was estimated using the half-sphere formula (Colony area = 2 × [(maximum diameter/2 × minimum diameter/2) × π]) following Lozano-Cortés & Berumen^24^. The projected surface area data was logarithmically transformed and from these data, size-frequency distributions were constructed^25^ and its skewness (g1) calculated^26^.

### Soft sediment macrobenthic survey

Sediment samples from 13 stations (Fig. 1) were collected across an inshore to offshore transect from Jazan to the Farasan Islands using a 0.1 m^2^ Van Veen grab (two replicates at each site) and sieved through a 1 mm mesh screen. A total of 14 soft sediment stations were sampled. Sites located in the inner shelf include JAZ2, JAZ3, JAZ4, JAZ6, JAZ7, JAZ8, FI1, FI2, FI3, FI4, FI5 while sites located in the outer shelf included FI6, FI7, FI8. Samples were preserved in 96% ethanol. Sediment sub-samples from each replicate were taken for grain-size analysis, organic matter content (loss on ignition) and metals. Conductivity Temperature Depth (CTD) vertical casts were carried out at each station. The CTD casts recorded conductivity, temperature, depth and oxygen of the water column. Water samples were also taken for nutrients and chlorophyll *a* from the surface and the bottom layers (see below).

In the laboratory, each sample was hand-sorted and then organisms identified using a stereomicroscope. Macrofauna were generally identified to the species level where possible or family level (see Supplementary Material). Sediment grain size was determined by wet sieving and calculation of dry weight percentage fractions following the Wentworth scale^27^. Grain size fractions were gravel (>2 mm), very coarse sand (<2 mm and >1 mm), coarse sand (<1 mm and >500 μm), medium sand (<500 μm and >250 μm), fine sand (<250 μm and >125 μm), very fine sand (<125 μm and >63 μm) and silt-clay (<63 μm). Loss on ignition (LOI) was measured by dry sediment loss after combustion at 450 °C (American Public Health Association 21^st^ Edn 2540 D + E Mod^28, 29^).

#### Water sample analysis

Water samples for chlorophyll *a* and nutrient quantification (NO_3_, NO_2_, PO_4_, SiO_2_) were taken at each station and/or reef. Water was collected from the surface in the case of coral reefs and from both the surface and bottom layers in sediment stations. Approximately 50 mL water was filtered through 0.22 μm membrane filters to collect nutrient samples which were frozen at −20 °C until analysis. To assess gradients in the primary productivity, surface water samples for chlorophyll *a* analysis were also taken in September 2014 and February 2015. For the analysis, 0.5–3 L of water were filtered through GF/F filters, wrapped in aluminum foil, and frozen at −20 °C on the research vessel and then to −80 °C (at the laboratory) until analysis. Chlorophyll *a* was extracted using 90% acetone^30, 31^. Following extraction, the raw fluorescence was measured with a Trilogy fluorometer (Turner Designs). Nutrient samples were analyzed using a Continuous Flow Analyzer (*SEAL AutoAnalyser 3 with XY2*/*3 Sampler*)^31^.

#### Data analysis

Hypotheses about changes in the structure and composition of benthic assemblages in the southern Red Sea were tested using a combination of multivariate and univariate techniques. Multivariate analyses were carried out with the statistical package PRIMER v6 and the PERMANOVA+ add-on package^32, 33^.

For coral reefs, shelf-position, depth, chlorophyll *a* and nutrients, sand, coralline algae and rock cover were used as potential explanatory variables. Nutrient and chlorophyll *a* data collected from the CTD casts were used in the analysis. Because nitrates and nitrites were highly correlated we used the nitrite measurements in subsequent statistical analyses where the percentage variation explained was highest. We analyzed the following univariate descriptors: number of taxa (OTUs), abundance (as % cover), Shannon-Wiener diversity, Margalef species richness and Pielou’s equitability. To visualize patterns of change in the composition and structure of the targeted assemblages across the shelf gradient, unconstrained (Principle Coordinates PCO) and constrained (distance-based redundancy analysis dbRDA^34^) ordination methods were applied. For reef datasets, the multivariate methods were based on Bray-Curtis similarities calculated from square-root transformed data. Benthic categories were related to the spatial patterns in composition and abundance of reef associated assemblages via Distance-based Linear Models (DistLM)^33^ where the most parsimonious model based on the ‘Best’ procedure and the R^2^ selection criterion was plotted using dbRDA. We also calculated the index of multivariate dispersion (MVDISP) based on Bray-Curtis similarity, Jaccard, Sorensen and Chi-Square distance.

The size frequency data was used to assess coral colony size differences between nearshore versus offshore reefs within the same coral genus using a non-parametric Mann–Whitney t-test. Kolgomorov-Smirnov test for two samples was carried out to compare the size-frequency distributions between the two types of reefs for each coral genus. These analyses were not carried out for the coral genus *Galaxea* due to the low number of colonies (only 2) found in the nearshore reefs. All the analyses were performed using the computer software Statistica 8.0.

Soft sediment macrobenthic community structure was characterized regarding abundance (*N*), total number of taxa (*S*), diversity (Shannon-Wiener index H’), and equitability (Pielou’s J’) indices. Ordination techniques, including Principle Component Ordination (PCO) were performed to better visualize the patterns of change in the macrobenthic assemblages. Distance-based linear modeling (DistLM) with step-wise and adjusted R^2^ selection criteria were performed to estimate the explained variation in macrobenthic assemblage structure by each environmental variable. The direction and magnitude of the relationship between environmental variables and assemblages (given by the vectors for each environmental variable) were visualized in distance-based redundancy analysis (dbRDA) biplots. Environmental data (depth, distance to the main shoreline, nutrients, and grain-size fractions) were normalized before the analyses, as they have different units and scales. For assemblages inhabiting sediments multivariate methods were also based on the Bray-Curtis similarities calculated from the square-root transformed values.

## Results

### Coral Reef Assemblages

**Figure 2:**
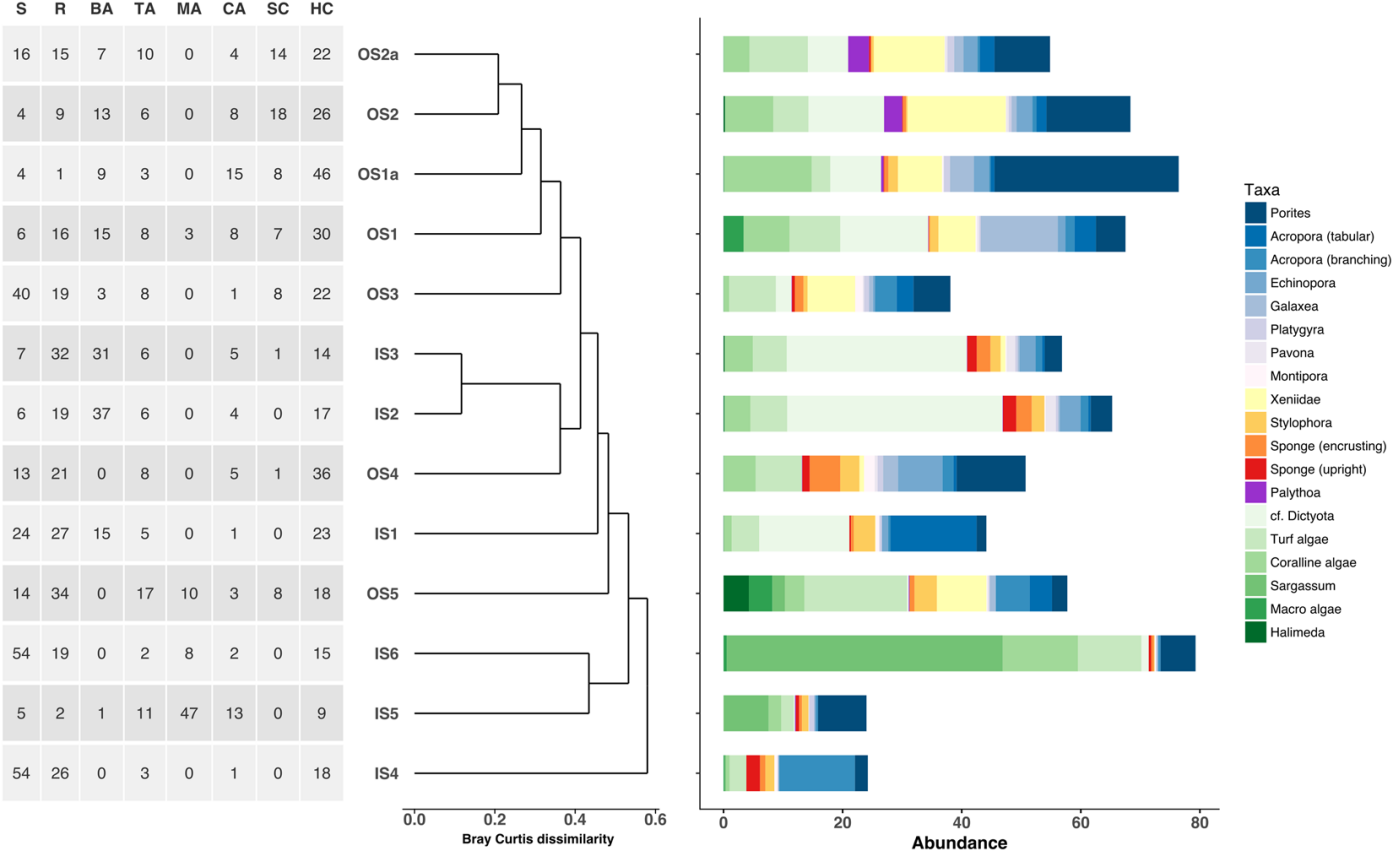
Cluster diagram of sites based on benthic composition of Bray-Curtis dissimilarity and associated benthic species by site indicating cross shelf gradient from Outer Shelf (OS) to Inner Shelf (IS). Note colors groupings on histogram where Blue = Corals, Red and Orange = Sponges, Green = Algae. S = Sand, R = Rubble, MA = Macroalgae, SC = Soft Coral, HC = Hard Coral, CA = Coralline Algae, BA = Brown Algae, TF = Turf Algae.

A total of 41 scleractinian OTUs were identified, along with 10 soft coral OTUs. *Porites* massive form was the consistently dominant reef-building coral across depth and shelf. Depending on the habitat, *Acropora* (three OTUs, *Acropora*, *Acropora* tabular and *Acropora* branching), *Echinopora*, *Stylophora*, *Galaxea* and *Pavona*, also accounted on average for more than 1% of the cover per reef. Scleractinian corals mainly dominated reefs in the outer shelf, while sand or macroalgae mainly dominated the inner shelf reefs. Inner shelf reefs harbored less abundant, less diverse benthic megafaunal assemblages than outer shelf reefs (Table 2). In general, the outer shelf reefs supported higher numbers of rare taxa (i.e. singletons+doubletons). An increase in the mean biological cover and number of taxa was also observed from 2–5 m to 8–10 m depth transects. The same pattern was detected for both hard and soft corals and to a lesser extent, coralline algae. The macroalgae/hard coral cover ratio was higher in inner shelf reefs compared to outer shelf reefs (Table 2). High levels of dominance characterized the inner shelf reefs but no clear pattern regarding depth was detected. Overall, 31 taxa were shared among all the groups of reefs (i.e., inner shelf 2–5 m and 8–10 m; outer shelf 2–5 m and 8–10 m). The outer shelf reefs supported 14 exclusive taxa, against one (*Padina*) in the inner shelf reefs (Fig. 2).

**Table 2:** Mean percentage (±SD) of benthic richness (S), cover (N), and coral and coraline algae cover in inner and outer-shelf reefs at both depth ranges.

Shelf location	Depth (m)	DS range (km)	No of reefs	S	N	HC cover	SC cover	CA cover	MA/HC ratio	Total S	Single-tons	Doubletons
Inner	2–5	15–32	4	17.6 ± 4.22	50.5 ± 25.53	16.4 ± 9.5	0.01 ± 0.05	4.4 ± 4.61	2.38	50	16	4
Inner	8–10	15.7	1	23.7 ± 1.53	63.0 ± 3.61	13.9 ± 4.6	0.8 ± 0.9	7.1 ± 2.22	3.1	33	11	6
Outer	2–5	74–104	4	27.4 ± 5.66	59.6 ± 13.95	25.6 ± 8.8	8.7 ± 6.6	4.3 ± 2.60	0.76	70	15	12
Outer	8–10	104–110	3	30.2 ± 5.12	73.2 ± 10.20	32.8 ± 12.8	9.6 ± 4.7	9.0 ± 5.15	0.78	64	15	11

Cluster ordination analyses performed on Bray-Curtis measures showed greater similarity in the outer reefs compared with inner shelf sites (Fig. 2). Overall, a consistent pattern emerged across the measures applied, i.e., lower heterogeneity in deeper than shallower transects and in outer than inner shelf reefs. The outer shelf reefs (particularly at the deeper transects) were mainly associated with higher cover of hard corals, soft corals and coralline algae, while most of the inner shelf reefs were associated with either upright sponges, cf. *Dictyota* or *Sargassum* (Fig. 2). For all the measures applied in the multivariate analyses, the MVDISP showed a consistent and increasing pattern, i.e., 2–5 m inner shelf >2–5 m outer shelf >8–10 m outer shelf >8–10 m inner shelf (Table 3).

**Table 3:** Results of the MVDISP routine. IS, Inner-shelf; OS, outer-shelf.

Groups	BC	Jaccard	Sorensen	Chi-square
7–10 m IS	0.057	0.06	0.06	0.133
7–10 m OS	0.534	0.623	0.623	0.565
<5 m OS	0.763	0.93	0.93	0.748
<5 m IS	1.336	1.2	1.2	1.333

#### Changes in size frequency and skewness

The corals surveyed ranged in diameter from 3 to 175 cm (Table 4). Statistically significant differences were detected between the size of the colonies from nearshore and offshore reefs for *Acropora*, *Echinopora* and *Porites* (Table 4). Smaller colonies were recorded in the nearshore reefs (Table 4). Juvenile coral density (mean ± SD) differed significantly between types of reefs for *Acropora* and *Echinopora* (p < 0.001) with lower densities recorded in the nearshore reefs (Table 4). The size-frequency distribution per coral genus was also statistically different for *Acropora* (p < 0.001) and *Echinopora* (p < 0.005) based on the Kolgomorov-Smirnov test. Only *Porites* showed negative values of skewness for the nearshore reefs. This more negatively-skewed size distribution is probably a result of the lack of abundant juveniles (<5 cm diameter, 58 in nearshore vs 234 in offshore reefs) and adult corals (>10 cm diameter, 34 in nearshore vs 415 in offshore) in the nearshore reefs in comparison with the offshore reefs.

**Table 4:** Coral abundance, size and density and skewness distribution for the four most abundant genera at the Farasan Islands, Southern Red Sea.

Coral	Reef type	N	Mean coral size (cm) ± SD	Coral size range (cm)	Coral density (ind/m^2^) ± SD	Skewness (g1)	MW t-test *p* value
*Acropora*	Nearshore	176	16.60 ± 9.74	3.91–58.62	1.16 ± 2.46	0.22	0.008
	Offshore	271	26.72 ± 21.06	5.10–174.82	1.27 ± 1.08	0.35	
*Echinopora*	Nearshore	58	16.26 ± 12.11	3.00–44.63	0.31 ± 0.78	0.37	0.015
	Offshore	100	26.47 ± 21.59	5.70–147.10	0.44 ± 0.44	0.20	
*Porites*	Nearshore	188	19.92 ± 12.55	2.87–61.62	1.07 ± 1.15	− 0.12	<0.001
	Offshore	649	23.56 ± 18.75	23.56–125.95	3.09 ± 1.03	0.23	
*Galaxea*	Nearshore	2	8.91 ± 1.30	7.99–9.84	0.01 ± 0.04	—	NA
	Offshore	575	10.84 ± 7.01	3.95–62.53	3.87 ± 7.07	—	

SD, Standard Deviation. Statistical differences between nearshore versus offshore reefs using the Mann-Whitney (MW) t-test for the most abundant genera.

#### Relationship between reef community patterns and environmental variables

**Figure 3:**
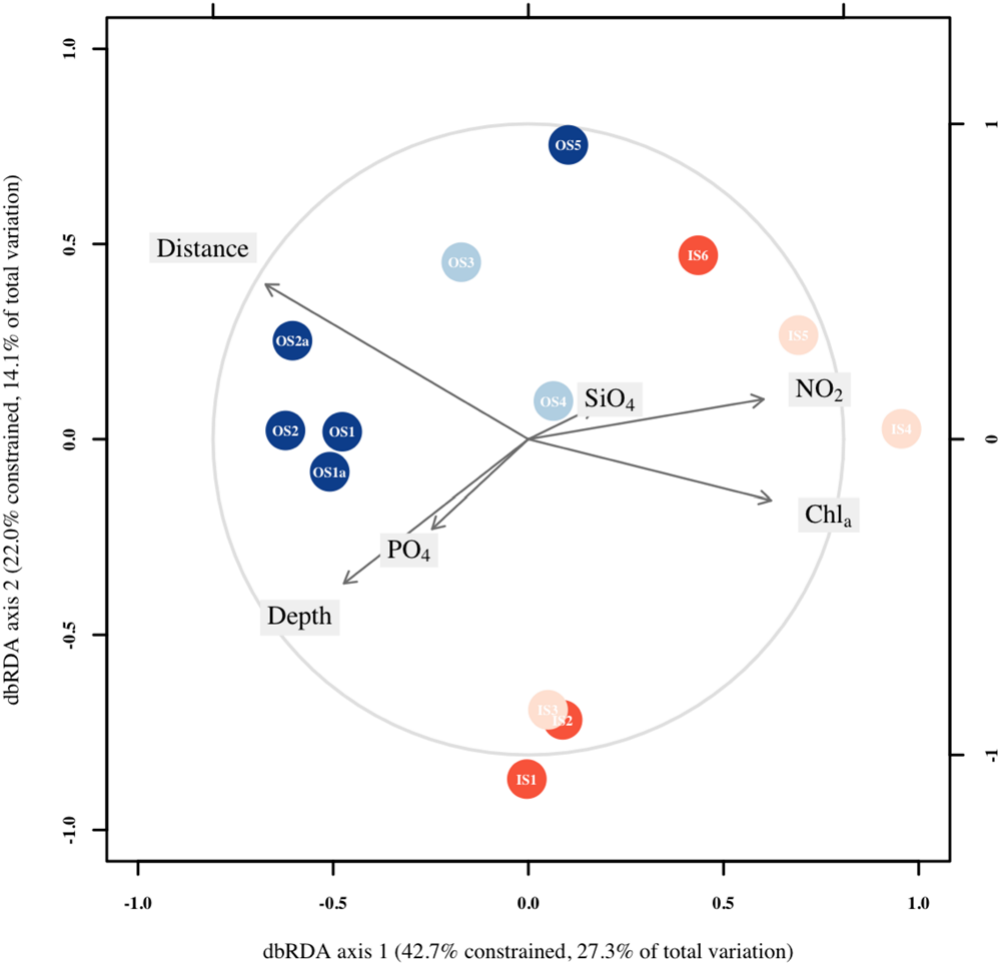
dbRDA based on coral reef survey benthic composition and associated environmental variables. Depth; Distance, distance to the main shoreline; SiO_2_, silicates; PO_4_, phosphates; NO_2_, nitrites; Chla, chlorophyll *a*.

Inshore reefs were associated with higher concentrations of nutrients (nitrites or phosphates) (Fig. 3). The distance to the main shoreline clearly separates both reef groups, suggesting a strong inshore to offshore pattern (Fig. 3). A number of environmental factors including nutrients, chlorophyll, depth and distance from the shore were significantly related to the composition of the benthic patterns (Table 5).

**Table 5:** Distance based linear modeling results based on coral reef survey benthic composition and associated environmental variables.

Variable	SS	Pseudo-*F*	*p* value
Distance	3073.6	3.22	0.001
Depth	1987.2	1.89	0.046
Phosphate	1125.0	0.99	0.408
Silicate	841.9	0.73	0.699
Nitrite	2439.3	2.41	0.009
Chlorophyll *a*	2503.7	2.49	0.007

### Soft sediment assemblages

#### Environmental patterns

The sampled area presents relatively high variability in terms of the environmental parameters analyzed. Depth varied from 5 m (JAZ6) to 138 m (FI5) and samples were distributed along an inshore to offshore gradient ranging from 2 to 127 km from the main shoreline (Table 6). Levels of phosphates (PO_4_) in the water were generally low (<0.1 μM) with only a few stations presenting values above 0.2 (FI5, FI7 and FI8). Variability was also high regarding the sediment particle size, with some stations, even within the same area, being either dominated by fine or sandy fractions (Table 6). This high variability of the sediment particle size between sites and the general pattern of a higher percentage of gravel and coarse sand in the coastal sites may explain why there was not a clear relationship between water column nutrient levels and organic content in the sediments for the nearshore JAZ sites.

**Table 6:** Results for the environmental variables analyzed in the sediment stations and from CTD casts.

Stations	Depth	DS	PO_4_	SiO_2_	NO_3_	NO_2_	Gravel	Sand	Silt-clay	LOI	Chl *a*
JAZ2	5.6	2.0	0.05	1.66	0.27	0.02	2.55	93.87	3.58	1.41	1.80
JAZ3	10.5	3.5	0.09	1.63	0.35	0.04	6.61	82.83	10.56	1.54	1.71
JAZ4	18.0	7.9	0.13	1.58	0.63	0.25	15.81	40.53	43.66	4.16	1.62
JAZ6	5.0	1.4	0.04	1.60	0.37	0.04	10.81	80.82	8.37	1.58	2.60
JAZ7	9.2	2.9	0.05	1.03	0.29	0.03	4.00	81.54	14.46	1.65	1.98
JAZ8	14.7	6.9	0.13	0.78	0.49	0.02	7.21	41.28	51.52	2.95	1.35
FI1	62.2	18.6	0.28	3.13	2.04	0.70	1.46	21.15	77.38	5.63	1.26
FI2	60.8	18.0	0.24	1.88	1.32	0.45	0.41	5.87	93.72	6.51	1.62
FI3	91.4	36.0	0.33	3.42	1.83	0.18	0.05	11.61	88.35	5.89	1.17
FI4	37.9	42.0	0.11	1.71	0.59	0.10	5.41	73.48	21.11	2.45	1.26
FI5	138.1	41.0	0.25	3.89	3.08	0.27	11.51	79.48	9.01	2.41	1.17
FI6	56.2	111.4	0.40	2.34	2.52	0.64	8.28	81.32	10.30	3.24	1.17
FI7	76.6	114.2	0.44	3.13	3.57	0.34	0.13	13.60	86.27	3.24	1.35
FI8	80.9	120.1	0.53	5.62	6.53	0.30	0.82	28.12	71.06	4.19	1.53

DS, distance to the main shoreline (km); nutrients (PO_4_, SiO_2_, NO_3_, NO_2_) concentrations in μM. Gravel, sand and silt-clay particles in percentage of the total sample. Loss on Ignition (LOI) in percentage. Surface Chlorophyll *a* (Chl *a*) measured in μg L^−1^ from CTD casts. Jazan City; JAZ; FI IS, Farasan Islands-Inner shelf; FI OS, Farasan Islands-Outer shelf.

#### Macrobenthic assemblages

**Figure 4:**
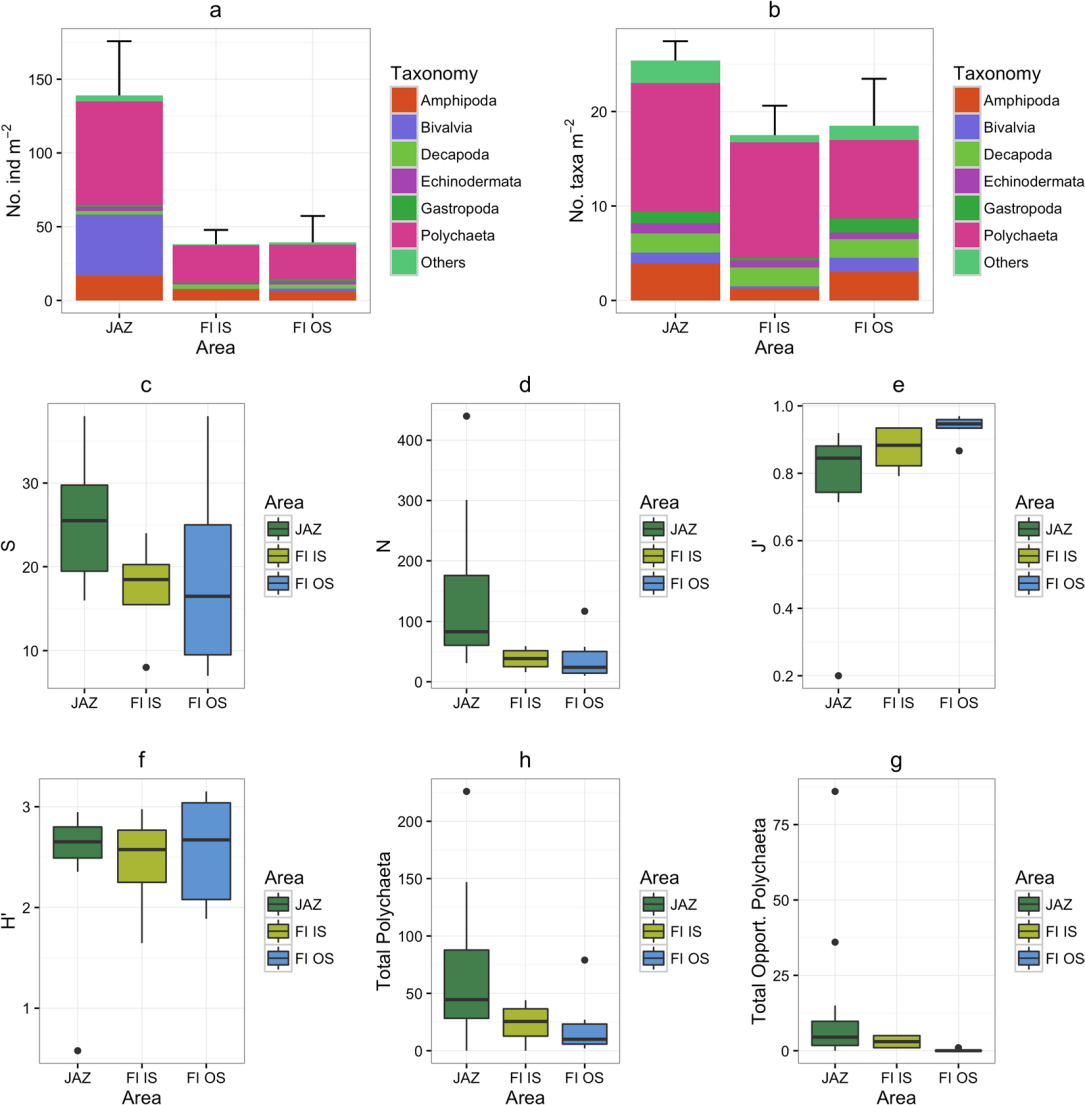
Relative abundance of the main soft sediment taxonomic groups in the different study areas (**a**) and relative proportion of the main taxonomic groups in terms of the number of taxa in the different study areas (**b**). Boxplots for the univariate variables number of taxa (S, taxa × 0.1 m^2^) (**c**), abundance (N, no. of individuals × 0.1 m^2^) (**d**), Pielou’s equitability (J’) (**e**) and Shannon-Wiener diversity (H’) (**f**), the total number of Polychaeta (**h**) and the total number of opportunistic Polychaeta (**g**) based on soft sediment macrofauna in the different areas. JAZ, Jazan; FI IS, Farasan Islands-Inner shelf; FI OS, Farasan Islands-Outer shelf.

In the subset of the samples selected, 154 taxa were identified. This number is highly underestimated, as Polychaeta, Decapoda and Echinodermata, some of the most abundant groups, were not identified to the genus or species level (family level for polychaetes and decapods; class level for echinoderms). Overall, Polychaeta (contributing from 49.5% to 65.8%, in Jazan and FI IS, respectively) and Amphipoda (contributing from 12.3% to 19.1%, in Jazan and FI IS, respectively) were the dominant groups in terms of abundance. Only at Jazan, bivalves (28% of the total abundance) were more abundant than amphipods (Fig. 4a). In terms of the number of taxa, Polychaeta was once more the dominant group across the study area, contributing to more than 45% (FI OS) of each area’s total number of taxa and reaching 69% in the FI IS (Fig. 4b). Amphipoda was in general the second highest contributor (7% to 14% of total number of taxa), followed by Decapoda (9% to 11% of total number of taxa) and Bivalvia (2% to 7% of total number of taxa) (Fig. 4b). When analyzed per area, Jazan was characterized by the highest number of taxa (25.5 ± 6.64), followed by FI IS and FI OS (Fig. 4c). Abundance was also higher in Jazan (Fig. 4d). In both cases, variability was high. The extremely high abundance values found in some of the replicates collected in the Jazan area (e.g., JAZ6 R1, 440 individuals), resulted in depressed equitability (J’) (Fig. 4e). Total polychaetes and opportunistic polychaetes were more abundant at the Jazan area, followed by the FI IS (Fig. 4g,h). Opportunistic polychaetes recorded included Capitellidae, *Cirratulus* sp., *Chaetozone* sp., *Notomastus* sp. amongst others. See Supplementary Table S1 for a full list of opportunistic polychaete species recorded by site.

**Figure 5:**
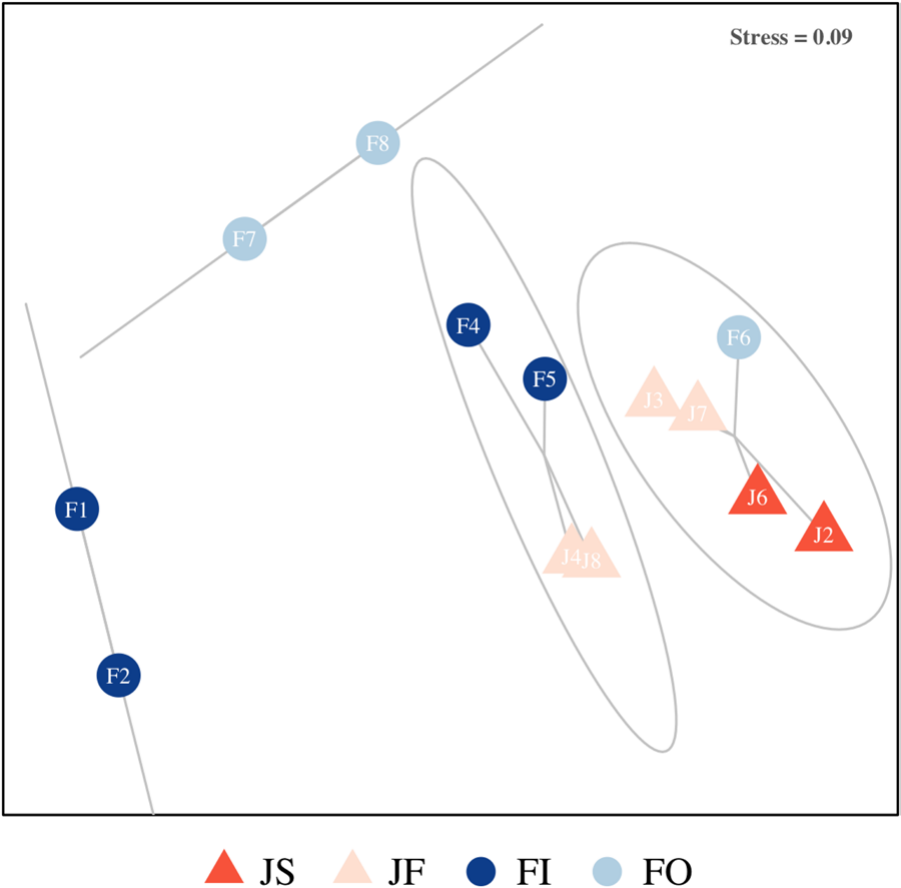
Principle component ordination (PCO) plots based on the Bray-Curtis similarity matrix after square-root transformation of the data from the different areas. JS, Jazan Shore; JF Jazan Far; FI Farasan Inner; FO Farasan Outer.

The multivariate analysis on the square-root transformed data identified four main groups (Fig. 5). Sites clearly grouped from inshore to offshore in the ordination. The near shore Jazan sites (JS: JAZ2, JAZ6) grouped together while sites further offshore from Jazan were grouped (JF: JAZ3, JAZ4, JAZ7, JAZ8). Similarly the Farasan Island soft sediment sites also clustered across the shelf with Farasan Inshore (FI; FI1, FI2, FI4, FI5) sites grouping together and Farasan Offshore sites grouping (FO; FI6, FI7, FI8). SIMPER analysis highlighted the higher abundance of bivalves and polychaetes (in general, but also the opportunistic Capitellidae and Cirratulidae) in Jazan and to a less extent in the FI IS area.

#### Relationship between biological patterns and environmental variables

**Figure 6:**
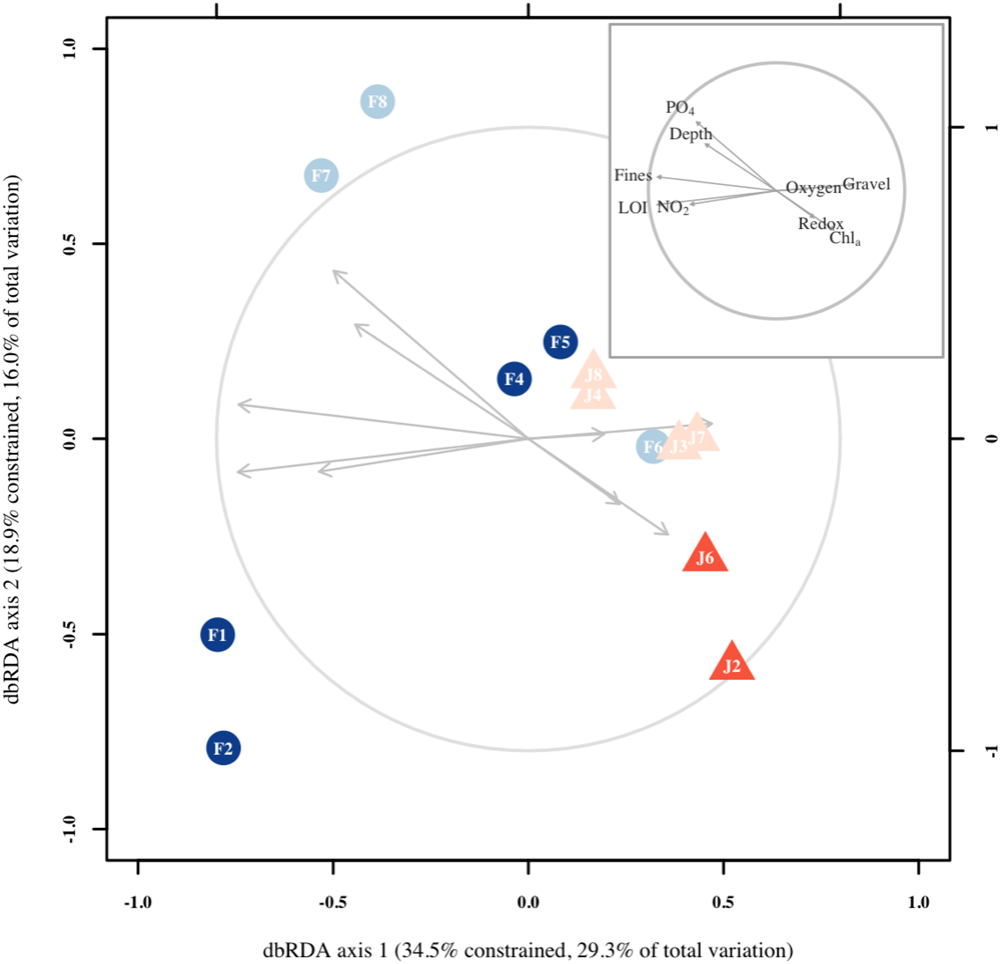
Distance-based redundancy analysis plot of square-root-transformed macrobenthic data with Bray-Curtis similarity index. Vectors are shown only for the most relevant environmental variables in the model. Length and direction of vectors indicate the strength and direction of the relationship. PO_4_, phosphates; NO_2_, nitrites; Chla, chlorophyll *a*; LOI, loss on ignition; Redox, reduction potential; Fines; >63 μm grain size fraction.

Distance-based linear modeling (DistLM) analysis showed that all environmental variables were significant for the adjusted R^2^ selection criterion. The sequential tests showed that a combination of variables that reflected nutrient enrichment (Chlorophyll *a*) and grain size (silt-clay, medium sand, and gravel) were significant to the adjusted R^2^ reduced model, which explained 20% of the total variation. The first two dbRDA axes explained 45% of the fitted variation. The dbRDA analysis separated near shore sites (JAZ2, JAZ6) from the Jazan area based on higher chlorophyll *a* content and reduced Redox potential (Fig. 6). Within the Farasan Islands, sediments at the inner shelf sites (FI1, FI2) presented higher levels of fine particles and also had higher organic contents within the sediments. Depth and phosphates were significantly related with the deeper sites at the offshore FI7 and FI8 sites (Fig. 6).

## Discussion

Cross shelf systems present the strongest persistent gradients in environmental conditions such as salinity, temperature, and nutrients in the marine environment and represent a transitional zone from coastal, estuarine and river influences to truly oceanic conditions^19^. The importance of cross shelf environmental conditions is reflected in the variation in diversity and composition of marine assemblages from in to offshore environments^20, 35^. Previous studies have reported distinct cross shelf distributional patterns for a range of taxonomic groups including larval fish, siphonophores, corals, sponges, soft corals, macroalgae, oysters and adult fish populations^36–43^.

This study investigates changes in the community composition of both coral reef and macrobenthic communities along a cross shelf gradient in the Farasan Islands and is, to our knowledge, the first synoptic study of soft sediment and reef diversity in the Red Sea region. We found clear cross shelf differences in community composition and structure for both reef and soft sediment habitats. Community composition of coral reef assemblages differed significantly in species composition, size distribution, and structure with location and depth while a clear inshore-offshore pattern of changing community composition and diversity was also observed for soft sediment communities. We consider these cross shelf biodiversity patterns in relation to key environmental factors including anthropogenic disturbance for both coral reef and soft sediment communities.

### Coral reef cross shelf patterns

Cross shelf biodiversity patterns for coral reef assemblages were recorded. Inner shelf reefs harbored less abundant and less diverse soft and hard coral assemblages with a higher macroalgae cover than outer shelf reefs. These cross shelf biodiversity patterns are in agreement with previously reported studies from the Red Sea^44^ and global studies^16, 45–47^. For example a number of studies have documented near shore sites that are dominated by macroalgae with relatively low hard coral cover compared to much higher coral cover and diversity at offshore sites^36, 48, 49^. The general trend is a loss of coral species diversity and a transition to dominance by macroalgal species. Such shifts from coral reef systems to alternate states (e.g., dominance of macroalgae, turf algae) as a result of multiple stressors have been documented worldwide^50^. Major stressors associated with the degradation of reefs have included pollution from nutrients^51^, sediments^52, 53^, fishing and altered trophic structures^54, 55^ as well as elevated sea surface temperatures^56–58^. These stressors can drive coral reef degradation directly, through increased coral mortality, or indirectly, by increasing susceptibility to coral diseases^59^. At longer time scales (decades), stress forces coral reef ecosystem degradation by reducing resiliency after disturbances, such as storms^60^ and bleaching events^61^. The combination of these processes has been the loss of coral reef health and the promotion of phase shifts to alternate ecological states^62^. In this study, there was also evidence of negatively skewed size-frequency distributions in nearshore coral populations associated with poor environmental conditions, a similar finding to other studies documenting reduced colony size in disturbed environments^63–65^.

We investigated the key environmental parameters influencing the observed coral shelf biodiversity patterns within the Red Sea, a region where nearly 60% of the reefs have been identified as at risk from anthropogenic activities^66^. Environmental factors correlated with coral diversity loss were related to elevated nutrient levels and distance from the shoreline. Distance from the shoreline included aspects of depth as well as changing levels of disturbance such as turbidity and enrichment. Variation in depth has been previously identified as a significant parameter in the diversity and distribution of various taxa including scleractinian corals^19^ and meiofaunal communities^67^. Higher nutrient availability and chlorophyll *a* concentrations at near shore sites were also associated with lower diversity of coral species and a higher dominance of algal cover. This trend indicates a potential impoverishment of these reef assemblages driven by human-activities in the nearshore region. This pattern supports literature findings that nearshore reefs, closest to terrestrial impacts, would show the greatest signs of degradation relative to reefs located in offshore areas. Previous studies have indicated that the main source of nutrients in the Red Sea come from the Indian Ocean, through the Gulf of Aden, which have a higher influence on the offshore locations due to the shelf bathymetry and oceanography circulation patterns on the shelf ^5, 68–72^. Higher nutrient concentrations were therefore expected in the offshore locations, however, measured nutrient and chlorophyll *a* concentrations found to be important factors influencing reef assemblages were higher in the inner shelf region. There has been the general perception that the flux of anthropogenic nutrient inputs relative to oceanic sources of nutrients such as upwelling is small and therefore anthropogenic inputs can have relatively little effect on the productivity of coastal waters. Alternatively recent studies investigating the relative role of anthropogenic nutrient sources from rival natural sources on small scales suggest that anthropogenic nutrients can provide a significant source of nitrogen for near shore productivity in coastal waters^73, 74^. Sources of anthropogenic disturbance and nutrient enrichment in the inner shelf region may occur due to the proximity to Jazan City, an urbanized area in the south of the Red Sea. The province of Jazan also has agricultural farming activity. Since, Jazan is located in a wadi area and is one of the locations with higher annual rainfall (131 mm/year) in Saudi Arabia^75^, surface run-off and storm water may therefore contribute to coastal nutrient enrichment in this region. This finding is similar to another Red Sea study^68^ that also predicted higher nutrient concentrations at offshore sites due to oceanographic sources of nutrients, but reported higher chlorophyll *a*, phosphorus, and particulate carbon concentrations at the near shore sites, attributed to a domestic wastewater treatment facility and industrial facilities. Further studies conducted in the Red Sea have identified land-based nutrient loading (particularly nitrogen levels) as a variable responsible for coral diversity loss or coral mortality^21, 76–78^. Numerous laboratory and field experiments^79–81^ have concluded that corals are negatively affected by increased levels of nutrients. Case studies of *in situ* observations demonstrate shifts from coral dominance to algal dominance and suggest linkages with chronic nutrient loading, including case studies in the Pacific Ocean^82, 83^, the Red Sea^84, 85^, the Caribbean^79, 86^, the Indian Ocean^87^ and the Atlantic Ocean^88^. As well as elevated nutrient enrichment in the coastal zone, there is also the potential for elevated turbidity in the near shore southern Red Sea. This is due to the wider coastal shelf in the southern region where sediments are less readily lost to deeper water. The shallow bathymetry created by these sediment deposits in the southern Red Sea combined with their fine nature results in substrate instability and associated turbidity in exposed conditions, which can limit the opportunity for the development of coral reefs. Currently there are few large-scale studies of water quality and biotic responses to increasing nutrients and turbidity from terrestrial runoff, and the links between reef health and water quality at larger scales have remained the subject of debate^89^. Our study demonstrates a loss of coral health and clear shifts from reef to macroalgae dominated communities at nearshore sites associated with potential anthropogenic nutrient enrichment. As noted above Jazan is in a flooding area with higher than normal precipitation rates for the country, which may contribute to higher levels of coastal nutrients from run-off. Fishing disturbance particularly from trawling activities can contribute to damage of corals. The relative role of other mechanisms that may also affect coral health including turbidity and depth that are captured by the variable ‘distance from the shore’ will also require further investigation.

### Soft sediment macrobenthic cross shelf patterns

This study also observed a clear inshore to offshore pattern of changing community composition and diversity for soft sediment macrobenthic communities. In contrast to coral reef patterns, the highest diversity and abundance of soft sediment communities were recorded at the inshore sites. Many studies have investigated spatial distribution patterns of macrobenthic communities in transitional estuarine systems and have found sediment composition, salinity, depth and organic content as relevant environmental factors explaining macrofaunal patterns^18, 90–92^. There are fewer comparable studies that have investigated cross shelf regional biodiversity patterns in coastal areas. Regional studies of the macrobenthic patterns from the Norwegian continental shelf identified environmental variables best correlated with macrofaunal communities, which included median grain size, silt-clay content of sediment and depth^93–95^. Relationships between the distribution of soft-bottom macrofauna and sediment characteristics, in general, have been studied for decades, and historically particle size has often been identified as an important variable explaining macrobenthic distribution^96–98^. It is also recognized that at any given location, a number of different interacting factors will be involved in explaining faunal patterns^98^. In this study, a combination of nutrient enrichment and grain size were identified as important factors affecting the ecology of the soft sediment macrobenthic communities. Sites closer to the shore (Jazan) were characterized by a higher number of bivalves and opportunistic polychaete species, as well as lower equitability, which may be indicative of mild disturbance. A peak of opportunistic species is often associated with mild disturbance such as organic enrichment as summarized in ref. 99 seminal review of the effects of organic enrichment and pollution on marine benthic communities. Currently, the high levels of diversity and abundance observed in the inshore Jazan area appear to support the hypothesis that the system is affected by mild enrichment. The region is not in a highly disturbed state where overall species richness is reduced and only high densities of small fast growing opportunistic species occur. In general, the macrofaunal species diversity recorded in this study was high, especially considering the taxonomic resolution of the study. Increasing the taxonomic resolution and expanding the spatial coverage of research in the Red Sea is likely to substantially increase the current number of macrofaunal taxa recorded supporting the overall high biodiversity and endemism previously reported for this region.

### Management implications

Globally, both terrestrial and aquatic ecosystems are experiencing declining biodiversity^100, 101^. This decline highlights the need to understand processes regulating diversity and the consequences of species loss for ecosystem function^102, 103^. Although recent studies have confirmed the existence of diversity gradients and investigated species richness patterns for numerous marine groups^23, 104^, the processes that shape these patterns have not been adequately resolved^105^. Bellwood & Hughes (2001) highlight that if we wish to protect global biodiversity, we must understand the processes that maintain diversity at these scales. The protection of habitat over large regional-scale areas has therefore been identified as a major goal for management strategies^105^. Achieving this outcome requires an improved understanding of the dynamics of reef and soft sediment ecosystems, of the processes that support or undermine resilience, and of the socio-economic drivers and governance systems that regulate water quality and rates of extraction of resources^50^.

There has been significant debate in the coral reef literature over the relative importance of environmental variables and stressors that regulate community structure and resilience. Human population growth and associated increases in nutrient loading^106^, destructive fishing^60^, compounded with ocean warming^58^ and stochastic environmental effects have been broadly debated to explain the increasing degradation of coral reefs worldwide. Discriminating among various stressors is critical to determine conservation strategies and to eventually ameliorate the accelerating degradation of coral reefs. In an attempt to differentiate between acute versus chronic stressors, several researchers have conducted broad correlative and statistical assessments of communities over large regional scales. These studies have suggested a clear interaction between eutrophication in conjunction with declining herbivorous organisms due to overfishing as direct causes for maintaining present undesirable phase shifts on coral reefs^107^. In this study, we identified factors that resulted in the loss of reef diversity and increases in opportunistic macrobenthic species influenced by anthropogenic disturbances including nutrient enrichment. Regional management strategies for coastal areas in the Red Sea therefore benefit from the active management of water quality and will also benefit from the protection of large reef and benthic areas.

In many parts of the world including the Kingdom of Saudi Arabia, water quality guideline values and targets have or are being developed as a tool to improve integrated coastal management (see ref. 108). A guideline value is defined as a numerical value of a water quality measure (e.g., light, nutrients, sediment, and agrochemicals) that will support and maintain the biological integrity of a water body and its benthic and pelagic ecosystems. Chlorophyll concentration is highly correlated with particulate nitrogen, particulate phosphorous, and suspended solids^53, 71^ and commonly used as a measure of water column productivity. High macroalgal cover is also widely accepted as an indicator of reef degradation, has been linked to high nutrient inputs and loss of herbivores, and causes mortality of corals and other reef and benthic organisms^109^. Nutrient threshold points (where increasing water column nutrients reach critical resilience levels such that they reduce recovery from phase shifts) have been widely postulated as approx. 1.0 um DIN^110–112^ for potential macroalgal overgrowth of coral-reef communities. Another useful tipping-point indicator is water column chlorophyll *a*, where levels in excess of 0.4–0.45 ug l^−1^ also indicate detrimental over-enrichment of nutrients^112, 113^. Within this study we recorded water column chlorophyll *a* levels up to 1.54 ug l^−1^ beyond the critical values reported in the literature for potential over enrichment effects requiring further assessment with other regions within the Red Sea.

Despite the extent of coral reefs in the Red Sea, the levels of biodiversity and endemism of the region are sorely understudied^6^. The lack of available information represents, in many cases, a significant hurdle for its conservation and management^6^. This study has identified changes in the biodiversity and community composition of both soft sediment macrofaunal and coral reef biodiversity at regional scales. These cross shelf patterns were related to nutrient enrichment and potential near shore anthropogenic disturbance providing important information for the management of marine coastal environments in the Red Sea.

## Electronic supplementary material

List of key taxonomic references and Table of numerical abundance of opportunistic Polychaete species by site
