# Supplementary material for: Cross shelf benthic biodiversity patterns in the Southern Red Sea
Source: Sci Rep. 2017 Mar 27;7:437. doi: 10.1038/s41598-017-00507-y (PMC5428672; doi:10.1038/s41598-017-00507-y)
Supplement: Supplementary file 1 — List of key taxonomic references and Table of numerical abundance of opportunistic Polychaete species by site [file 41598_2017_507_MOESM1_ESM.pdf]

Supplementary Material for:

Cross shelf benthic biodiversity patterns in the Southern Red Sea

Joanne Ellis[1*], Holger Anlauf[1], Saskia Kürten[1], Diego Lozano-Cortés[2], Zahra Alsaffar[1], Joao Cúrdia[1], Burton Jones[1], Susana Carvalho[1]

[1]Red Sea Research Center, Division of Biological and Environmental Science and Engineering, King Abdullah University of Science and Technology, Thuwal 23955-6900, Kingdom of Saudi Arabia

[2]Environmental Protection Department, Saudi Aramco, Dhahran 31311, Kingdom of Saudi Arabia

Supplementary Taxonomic References: Main list of references used to identify macrobenthic organisms from the Saudi Arabian Red Sea

Polychaeta

Amoureux, L., Annélides polychètes du Golfe d'Aqaba (Mer Rouge). Description d'un genre nouveau et de deux espèces nouvelles. *Bulletin du Muséum National d'Histoire Naturelle, Paris, 4e série, Section A* **5 (3)**, 723-742 (1983).

Amoureux, L., Rullier, F., Fishelson, L., Systématique et écologie d'annélides polychètes de la presqu'Il du Sinai. *Israel Journal of Zoology* **27**, 57-163 (1978).

Ben-Eliahu, M.N., Polychaete cryptofauna from rims of similar intertidal vermetid reefs on the Mediterranean coast of Israel and in the Gulf of Elat: Nereidae (Polychaeta Errantia). *Israel Journal of Zoology* **24**, 177-191 (1975).

Ben-Eliahu, M. N., Polychaete cryptofauna from rims of similar intertidal vermetid reefs on the Mediterranean coast of Israel and in the Gulf of Elat: Serpulidae (Polychaeta Sedentaria). *Israel Journal of Zoology* **25**, 103-119 (1976*a*).

Ben-Eliahu, M.N., Polychaete cryptofauna from rims of similar intertidal vermetid reefs on the Mediterranean coast of Israel and in the Gulf of Elat: Sedentaria. *Israel Journal of Zoology* **25**, 121-155 (1976*b*).

Ben-Eliahu, M.N., Polychaete cryptofauna from rims of similar intertidal vermetid reefs on the Mediterranean coast of Israel and in the Gulf of Elat: Syllinae and Eusyllinae (Polychaeta Errantia: Syllidae). *Israel Journal of Zoology* **26**, 1-58 (1977*a*).

Ben-Eliahu, M.N., Polychaete cryptofauna from rims of similar intertidal vermetid reefs on the Mediterranean coast of Israel and in the Gulf of Elat: Exogoninae and Autolytinae (Polychaeta Errantia: Syllidae). *Israel Journal of Zoology* **26**, 59-99 (1977*b*).

Böggemann, M., Revision of the Glyceridae Grube 1850 (Annelida: Polychaeta). *Abhandlungen der Senckenbergischen Naturforschenden Gesellschaft, Frankfurt am Main* **555**, 1-249 (2002).

Böggemann, M., Revision of the Goniadidae (Annelida, Polychaeta). *Abhandlungen des Naturwissenschaftlichen Vereins in Hamburg* (NF) **39**, 1-354 (2005).

Carrera-Parra, L.F., Phylogenetic analysis of Lumbrineridae Schmarda, 1861 (Annelida: Polychaeta). *Zootaxa* **1332**: 1-36 (2006).

Darbyshire, T., Mackie, A.S.Y., Species of *Litocorsa* (Polychaeta: Pilargidae) from the Indian Ocean and South China Sea. *Hydrobiologia* **496**, 63-73 (2003).

Day, J.H, Israel South Red Sea Expedition, 1962, Reports. No. 7. Some Polychaeta from the Israel South Red Sea Expedition, 1962. *Sea Fisheries Research Station Bulletin, Haifa* **38**, 15-27 (1965).

Day, J.H., A monograph on the polychaeta of Southern Africa. Part I. Errantia. Part II. Sedentaria. *Trustees of the British Museum (Natural History), London*, 656: xxix + xvii (2) + 1-878 (1967).

Fauchald, K., The polychaete worms. Definitions and keys to the orders, families and genera. *Natural History Museum of Los Angeles County, Science Series* **28**, 1-188 (1977).

Fauchald, K., A review of the genus *Eunice* (Polychaeta: Eunicidae) based upon type material. *Smithsonian Contributions to Zoology* **523**, 1-422 (1992).

Fauvel, P., Annélides polychètes nouvelles de l'Afrique Orientale. *Bulletin du Muséum National d'Histoire Naturelle, Paris* **24 (7)**, 503-509 (1918).

Fauvel, P., Annelida Polychaeta. *The Fauna of India including Pakistan, Ceylon, Burma and Malaya*. The Indian Press, Allahabad, 507 pp., 1 map. (1953).

Fitzhugh, K., A systematic revision of the Sabellidae-Caobangiidae-Sabellongidae complex (Annelida: Polychaeta). *Bulletin of the American Museum of Natural History* **192**, 1-104 (1989).

Gil, J., *The European Fauna of Annelida Polychaeta*. PhD Thesis. Universidade de Lisboa, Faculdade de Ciências, Departamento de Biologia Animal. xlii + 1554 pp (2011).

Gravier, C. Contribution à l'étude des annélides polychètes de la Mer Rouge. Première partie. *Nouvelles Archives du Museum d'Histoire Naturelle de Paris, 4e Série* **2**, 137-282, pls. 9-14 (1900*a*).

Gravier, C., Sur un type nouveau de Syllidien, *Fauvelia* (nov.gen.) *Martinensis* (n.sp.). *Bulletin du Muséum d'Histoire Naturelle, Paris* **6**, 371-374 (1900*b*).

Gravier, C., Contribution à l'étude des annélides polychètes de la Mer Rouge (suite). *Nouvelles Archives du Muséum d'Histoire Naturelle de Paris, 4e Série* **3**, 147-268, plates 7-10 (1902).

Gravier, C., Sur les annélides polychètes de la Mer Rouge (Serpulides). *Bulletin du Muséum d'Histoire Naturelle, Paris* **12**, 110-115 (1906*a*).

Gravier, C., Contribution à l'étude des Annélides Polychètes de la Mer Rouge (suite). *Nouvelles Archives du Muséum d'Histoire Naturelle, Paris, 4e Série* **8**, 123-236, plates 1-8 (1906*b*).

Gravier, C., Contribution à l'étude des Annélides Polychètes de la Mer Rouge (suite). *Nouvelles Archives du Muséum d'Histoire Naturelle, Paris, 4e Série* **10**, 67-168 (1908).

Hartmann-Schröder, G., Polychaeten aus dem Roten Meer, *Kieler Meeresforschungen* **16 (1)**, 69-125 (1960).

Holthe, T., Evolution, systematics, and distribution of the Polychaeta Terebellomorpha, with a catalogue of the taxa and a bibliography. *Gunneria* **55**, 1-236 (1986*a*).

Holthe, T., Polychaeta Terebellomorpha. *Marine Invertebrates of Scandinavia* **7**, 1-192 (1986*b*).

Hutchings, P., Glasby, C., Description of the widely reported terebellid polychaetes *Loimia medusa* (Savigny) and *Amphitrite rubra* (Risso). *Mitteilungen des Hamburgischen Zoologischen Museums und Instituts* **92** (Ergbd.), 149-154 (1995).

Hylleberg, J., Nateewathana, A., Polychaetes of Thailand. Nereididae (part 2): *Ceratocephale* and *Gymnonereis*, with description of two new species and notes on the subfamily Gymnonereidinae. *Phuket Marine Biological Center, Research Bulletin* **49**, 1-20 (1988).

Licher, F., Revision der Gattung *Typosyllis* Langerhans, 1879 (Polychaeta: Syllidae). Morphologie, Taxonomie und Phylogenie. *Abhandlungen der Senckenbergischen Naturforschenden Gesellschaft* **551**, 1-336 (1999).

Mortimer, K., Cassà, S., Martin, D., Gil, J., New records and new species of Magelonidae (Polychaeta) from the Arabian Peninsula, with a re-description of *Magelona pacifica* and a discussion on the magelonid buccal region. *Zootaxa* **3331**: 1-43 (2012).

Muir, A.I., Bamber, R.N., New polychaete (Annelida) records and a new species from Hong Kong: the families Polynoidae, Sigalionidae, Chrysopetalidae, Pilargiidae, Nereididae, Opheliidae, Ampharetidae and Terebellidae. *Journal of Natural History* **42 (9-10)**, 797-814 (2008).

Nygren, A., Revision of Autolytinae (Syllidae: Polychaeta). *Zootaxa* **680**, 1-314 (2004).

Pancucci-Papadopoulou, M.A., Murina, G.V., Zenetos, A., The phylum Sipuncula in the Mediterranean Sea. *Monographs on Marine Science* **2**, 1-109 (1999).

Pettibone, M.H., Revision of the Pilargidae (Annelida: Polychaeta), including descriptions of new species, and redescripton of the pelagic *Podarmus ploa* Chamberlin (Polynoidae). *Proceedings of the United States National Museum* **118 (3525)**, 155-207 (1966).

Pettibone, M.H., Revision of the aphroditoid polychaetes of the family Eulepethidae Chamberlin (= Eulepidinae Darboux; = Pareulepidae Hartman). *Smithsonian Contributions to Zoology* **41**, 1-44 (1969).

Pettibone, M.H., Additions to the Family Eulepethidae Chamberlin (Polychaeta: Aphroditacea). *Smithsonian Contributions to Zoology* **441**, 1-51 (1986).

Pettibone, M.H., Revision of the aphroditoid polychaetes of the Family Acoetidae Kinberg (= Polyodontidae Augener) and reestablishment of *Acoetes* Audouin & Milne-Edwards, 1832, and *Euarche* Ehlers, 1887. *Smithsonian Contributions to Zoology* **464**, 1-138 (1989).

Pleijel, F., Phylogeny and classification of the Phyllodocidae (Polychaeta). *Zoologica Scripta* **20 (3)**, 225-261 (1991).

Pleijel, F., Polychaeta Phyllodocidae. *Marine Invertebrates of Scandinavia* **8**, 1-158 (1993).

San Martín, G., Annelida Polychaeta II. Syllidae. *Fauna Ibérica* **21**, 1-554 (2003).

Storch, V., Drei neue polychaeten aus dem litoral des Roten Meeres. *Kieler Meeresforschungen* **22 (2)**, 171-175, 2 plates (1966).

Storch, V., Neue Polychaeten aus der Sandfauna des Roten Meeres. *Zoologischer Anzeiger*, **178 (1/2)**, 102-110 (1967).

Strelzov, V.E., *Polychaete worms of the family Paraonidae Cerruti, 1909 (Polychaeta, Sedentaria)*. Akademiya Nauk SSSR, Leningrad, 1-170, plates 1-9 in Russian; also 1979, translation and publication of the Russian original by Amerind Publishing Co. Pvt. Ltd., New Delhi, 212 pp., for the Smithsonian Institution, and the National Science Foundation, Washington (1973).

Wehe, T., Fiege, D., Annotated checklist of the polychaete species of the seas surrounding the Arabian Peninsula: Red Sea, Gulf of Aden, Arabian Sea, Gulf of Oman, Arabian Gulf. *Fauna of Arabia* **19**, 7-238 (2002).

Wehe, T., Revision of the scale worms (Polychaeta: Aphroditoidea) occurring in the seas surrounding the Arabian Peninsula. Part I. Polynopidae. *Fauna of Arabia* **22**, 23-197 (2006).

Wehe, T., Revision of the scale worms (Polychaeta: Aphroditoidea) occurring in the seas surrounding the Arabian Peninsula. Part II. Sigalionidae. *Fauna of Arabia* **23**, 41-124 (2007).

Wesenberg-Lund, E., Polychaetes of the Iranian Gulf. *Danish Scientific Investigations in Iran* **4**, 247-400 (1949).

Willey, A., Report on the Polychaeta collected by Professor Herdman, at Ceylon, in 1902. *Report to the Government of Ceylon on the Pearl Oyster Fisheries of the Gulf of Manaar, by W.A. Herdman, D.Sc., F.R.S., P.L.S., with supplementary reports upon the Marine Biology of Ceylon, by Other Naturalists. Part IV. Supplementary Report* **30**, 243-324 8 plates (1905).

Crustacea, Mollusca and Echinodermata

Apel, M., Spiridonov, V.A., Taxonomy and zoogeography of the portunid crabs (Crustacea: Decapoda: Brachyura: Portunidae) of the Arabian Gulf and adjacent waters, in Krupp, F. Mahnert, V. (eds) *Fauna of Arabia* **17**, 159–331 (1998).

Bellan-Santini, D., Karaman, G., Krapp-Schickel, G., Ledoyer, M., Myers, A.A., Ruffo, S., Schiecke, U., The Amphipoda of the Mediterranean. Part 1. Gammaridae (Acanthonotozomatidae to Gammaridae). *Mémoires de l'Institut Océanographique (Monaco)* **13**, 1-364 (1982).

Bellan-Santini, D., Diviacco, G., Krapp-Schickel, G., Myers, A.A., Ruffo, S., The Amphipoda of the Mediterranean. Part 2. Gammaridea (Haustoriidae to Lysianassidae). *Mémoires de l'Institut Océanographique (Monaco)* **13**, 365-576 (1989).

Bellan-Santini, D., Karaman, G., Krapp-Schickel, G., Ledoyer, M., Ruffo, S., The Amphipoda of the Mediterranean. Part 3. Gammaridea (Melphidippidae to Talitridae), Ingolfiellidea, Caprellidea. *Mémoires de l'Institut Océanographique (Monaco)* **13**, 577-813 (1993).

Chace, F.A., Jr., The Caridean Shrimps (Crustacea: Decapoda) of the Albatross Philippine Expedition, 1907-1910, Part 2: Families Glyphocrangonidae and Crangonidae. Series - Smithsonian Contributions to Zoology, No 397. Smithsonian Institution Press, Washington, USA. 63 pp. (1984).

Chace, F.A., Jr., The Caridean Shrimps (Crustacea: Decapoda) of the Albatross Philippine Expedition, 1907-1910, Part 5: Family Alpheidae. Series - Smithsonian Contributions to Zoology, No 466. Smithsonian Institution Press, Washington, USA. 99 pp. (1988).

Chace, F.A., Jr., Bruce, A.J., The Caridean Shrimps (Crustacea: Decapoda) of the Albatross Philippine Expedition, 1907-1910, Part 6: Superfamily Palaemonoidea. Series - Smithsonian Contributions to Zoology, No 543. Smithsonian Institution Press, Washington, USA. 152 pp. (1993).

Chace, F.A., Jr., The Caridean Shrimps (Crustacea: Decapoda) of the Albatross Philippine Expedition, 1907-1910, Part 7: Families Atyidae, Eugonatonotidae, Rhynchocinetidae, Bathypalaemonellidae, Processidae, and Hippolytidae. Series - Smithsonian Contributions to Zoology, No 587. Smithsonian Institution Press, Washington, USA. 106 pp. (1997).

Clark, A.M., Rowe, F.W.E., Monograph of Shallow-Water Indo-West Pacific Echinoderms. Trustees of the British Museum (Natural history), London, England. 238 pp. (1971).

De Bruyne, R.H., The Complete Encyclopedia of Shells. Rebo Publishers, Lisse, The Netherlands. 336 pp. (2003).

De Freitas, A.J., The Penaeoidea of Southeast Africa. I. The Study area and key to the southeast African species. Investigational Report No 56. The oceanographic research Institute, Durban, South Africa. 31 pp. (1984).

Gutu, M., Contributions to the knowledge of the genus *Apseudes* Leach, 1814 (Crustacea: Tanaidacea, Apseudomorpha) from the Mediterranean Basin and North African Atlantic. *Travaux du Muséum National d'Histoire Naturelle Grigore Antipa* **44**, 19-39 (2002).

Haig, J., Sur une collection de crustacés porcellanes (Anomura: Porcellanidae) de Madagascar et des Comores. *Cahiers ORSTOM - Série Océanographie* **3 (4)**, 39-50 (1965).

Herbert, D.G., An annotated catalogue and bibliography of the taxonomy, synonymy and distribution of the Recent Vetigastropoda of South Africa (Mollusca). *Zootaxa* **4049**, 1-98 (2015).

Huber, M., Compendium of Bivalves. ConchBooks, Hackenheim, Germany. 901 pp. (2010).

Huber, M., Compendium of Bivalves 2 - A full color guide to the Remaining Families. ConchBooks, Hackenheim, Germany. 907 pp. (2015).

Janssen, R., Zuschin, M., Baal, C., Gastropods and their habitats from the northern Red Sea (Egypt: Safaga) - Part 2: Caenogastropoda: Sorbeoconcha and Littorinimorpha. *Annalen des Naturhistorischen Museums in Wien*, Serie A **113**, 373–509 (2011).

Keable, S.J, Taxonomic Revision of Natatolana (Crustacea: Isopoda: Cirolanidae). *Records of the Australian Museum* **58**, 133–244 (2006).

McLaughlin, P.A., Illustrated keys to families and genera of the superfamily Paguroidea (Crustacea: Decapoda: Anomura), with diagnoses of genera of Paguridae, in Lemaitre, R., Tudge, C.C. (eds), Biology of the Anomura. Proceedings of a symposium at the Fifth International Crustacean Congress, Melbourne, Australia, 9–13 July 2001. *Memoirs of Museum Victoria* **60(1)**, 111-144 (2003).

Naderloo, R., Sari, A., Iranian Subtidal Leucosiid Crabs (Crustacea: Deacapoda: Brachyura) of the Persian Gulf: Taxonomy and Zoogeography. *Iranian Journal of Animal Biosystematics* **1(1)**, 28-43 (2005).

Naderloo, R., Türkay, M., Apel, M., Brachyuran crabs of the family Macrophthalmidae Dana, 1851 (Decapoda: Brachyura: Macrophthalmidae) of the Persian Gulf. *Zootaxa* **2911**, 1-42 (2011).

Öztürk, B., Scaphopod species (Mollusca) of the Turkish Levantine and Aegean seas. *Turkish Journal of Zoology* **35(2)**, 199-211 (2011).

Petrescu, I., Cumacea. Travaux du Muséum National d'Histoire Naturelle *Grigore Antipa* **38**, 115-175 (1997).

Sakai, K., Axioidea of the World and a Reconsideration of the Callianassoidea (Decapoda, Thalassinidea, Callianassida). Crustaceana Monographs, 13. Brill, Leiden, Boston, USA. 616 pp. (2011).

Sakai, K., Türkay, M., A review of the collections of the Infraorders Thalassinidea Latreille, 1831 and Callianassidea Dana, 1852 (Decapoda, Pleocyemata) lodged in three German museums, with revised keys to the genera and species. *Crustaceana* **87**, 129-211 (2014).

Scarabino, V., New species and new records of scaphopods from New Caledonia, in Héros, V., Cowie, R. H., Bouchet, P. (eds), Tropical Deep-Sea Benthos 25. *Mémoires du Muséum national d'Histoire naturelle* **196**, 215-268 (2008).

Slieker, F.J.A., Chitons of the World – an illustrated synopsis of recent Polyplacophora. L'informatore Piceno, Ancora, Italy. 154 pp. (2000).

Vereshchaka, A.L., Olesen, J., Lunina, A.A., Global Diversity and Phylogeny of Pelagic Shrimps of the Former Genera *Sergestes* and *Sergia* (Crustacea, Dendrobranchiata, Sergestidae), with Definition of Eight New Genera. *PLoS ONE* **9 (11)**, 1-32 (2014).

Wooldridge, T.H., Victor, R., Additions to the mysid fauna (Crustacea: Mysidacea) from coastal waters of Oman, including descriptions of two new species. *Hydrobiologia* **511**, 247-258 (2004).

Zuschin, M., Janssen, R., Baal, C., Gastropods and their habitats from the northern Red Sea (Egypt: Safaga) - Part 1: Patellogastropoda, Vetigastropoda and Cycloneritimorpha. *Annalen des Naturhistorischen Museums in Wien*, *Serie A* **111**, 73-158 (2011).

Supplementary Table S1: Numerical abundance of opportunistic Polychaete species by site. Where JAZ = Jazan and FI = Farasan

Species	JAZ2	JAZ3	JAZ4	JAZ6	JAZ7	JAZ8	FI1	FI2	FI3	FI4	FI5	FI6	FI7	FI8
cf. *Capitellethus* sp.	3	5	1	7	7						2			
Mediomatus sp.											1			
Notomastus sp.				1										
cf. Pseudoleiocapitella sp.				1										
Scyphoproctus cf. *pullielloides*	38	8			86						1			
Capitellidae n.id.	2											1		
Pulliella sp.														
cf. Leiocapitella					1									
Mesochaetopterus sp.		1										8		
Chaetopterus sp.												1		
Bhawania cryptocephala												1		
Caulleriella sp.	3				1									
Chaetozone sp.1	1	4			3	1								
Chaetozone sp.2					3						1			
Cirratulus sp.					1									
Monticellina sp.		4	4	1	1	2					1	1		
